# Comparative Assessment of Fatty Acids in Clams From Capo Peloro Lagoon (Sicily)

**DOI:** 10.1002/fsn3.70933

**Published:** 2025-09-12

**Authors:** Salvatore Giacobbe, Giuseppa Di Bella, Benedetta Sgrò, Fabio Scarpa, Daria Sanna, Ilenia Azzena, Marco Casu, Federica Litrenta, Vincenzo Lo Turco

**Affiliations:** ^1^ Department of Chemical, Biological, Pharmaceutical and Environmental Sciences (CHIBIOFARAM) University of Messina Messina Italy; ^2^ Department of Biomedical and Dental Sciences and of Morphological and Functional Images (BIOMORF) University of Messina Messina Italy; ^3^ Department of Biomedical Sciences University of Sassari Sassari Italy; ^4^ Department of Veterinary Medicine University of Sassari Sassari Italy

**Keywords:** clams, fatty acids content, health lipid indices, lagoons, statistical analysis

## Abstract

In Italy, the consumption and the production of shellfish, including clams, is considerable and has a significant impact on human health. In the lagoon of Capo Peloro (Sicily), three native clam species, *Cerastoderma glaucum* , *Ruditapes decussatus*, and *Polititapes aureus*, are traditionally farmed, locally commercialized, and highly appreciated by consumers. The fatty acid (FA) profile of these Sicilian species collected in 2023 and 2024 was evaluated and compared with the non‐commercial samples from the reserve of Oliveri‐Tindari (Sicily) and the extensively commercial *R. decussatus* from Sardinia. The results confirmed a low‐fat product, rich in C16:0 (22.37%–31.88%) and DHA (9.59%–17.48%). Samples from Capo Peloro are characterized by the highest content of saturated FAs (42.78%–50.88%) while samples from Sardinia have the highest content of polyunsaturated FAs (37.26%–40.83%). However, the fat in all samples demonstrated high nutritional quality, supported by good nutritional indexes and significant levels of EPA and DHA. Statistical analysis, used to differentiate the samples according to location, sampling year, and species, highlights the influence of environmental factors, seasonal variation, and genetic diversity on the FA composition. Among these, climatic temperature appears to be the most influential factor in regulating the balance between saturated and unsaturated FAs.

## Graphical abstract

The total fat content and fatty acid profile were evaluated in three species of edible clam (R. decussatus, P. aureus and C. glaucum) from the Capo Peloro and Oliveri‐Tindari lagoons in Sicily, as well as in R. decussatus from two Sardinian lagoons. All species exhibited a high saturated fatty acid content, a good level of DHA, and high‐quality lipids. The year of sampling has the greatest influence on the fatty acid profile of clams.

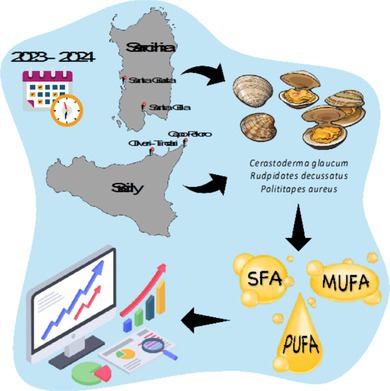

## 1 Introduction

Fishes and shellfishes are nutrient‐rich food sources that are consumed primarily by people who live in coastal areas (Prato et al. 2019). The Mediterranean diet proposes a moderate consumption of seafood, considering it a good source of protein and lipids (Davis et al. 2015).

Among shellfish, bivalve mollusks mostly used as a food source in human diets are clams, mussels, oysters and scallops. Italy, the country symbol of the Mediterranean diet, is an important producer and consumer of bivalve, having in the “Spaghetti alle vongole” (spaghetti with clams) one of the most consumed and appreciated traditional dishes (Durazzo et al. 2017).

The FAO estimated a production of 115,580 t against a consumption of 5.08 kg per person for 2021 (FAO 2022). The main bivalve mollusks farmed are mussels, *Mytilus galloprovincialis* (Lamarck, 1819), and clams, *Ruditapes philippinarum* (A. Adams & Reevw, 1850) (Tamburini et al. 2022).

The consumption of clams has a strong influence on human health, as they contain bioactive compounds and high‐quality nutrients, such as protein with high biological value, polyunsaturated fatty acids (PUFAs), mainly of the n‐3 series, such as eicosapentaenoic acid (EPA, C20:5 n‐3), docosahexaenoic acid (DHA, C22:6 n‐3), vitamins, and essential minerals, such as calcium and iron (Moniruzzaman et al. 2021). Among PUFAs, EPA is the precursor of a family of prostaglandins that regulate blood clotting, while DHA plays a crucial role in the function and development of the brain and reproductive system. The assumption through the diet of both EPA and DHA also decreases blood triglycerides, the risk of cardiovascular disease, and blood pressure, and prevents cardiac arrhythmias and sudden death (Gammone et al. 2019). EPA and DHA also have beneficial effects on other health issues such as skin diseases, inflammatory bowel disease, and cancer (Yang et al. 2020).

According to LARN (Reference Intake of Nutrients and Energy for Italian Population), the Adequate Intake (AI) of EPA + DHA should be 250 mg/day and the Recommended Intake (RI) of PUFAs, PUFA n‐6, and PUFAs n‐3 should vary between 5%–10%, 4%–8%, and 0.5%–2.0% of the total daily energy intake, respectively, as part of a balanced and healthy diet for an adult (Italian Society of Human Nutrition, SINU 2014). Moreover, the dietary recommendation of EFSA (European Food Safety Authority) for EPA and DHA suggests an intake between 250 and 500 mg/day for adults in order to reduce the cardiovascular risk (EFSA Panel on Dietetic Products, Nutrition and Allergies (NDA) 2012).

However, the quality and availability of clams can also vary widely because it depends mainly on seasonality and geographic location (Wright et al. 2018). The fatty acids are influenced by seasonal changes related to the characteristics of the species and the quality of the aquatic environment (i.e., availability of nutrients and mineral elements, reproductive cycle, diet, climatic conditions and, sediment) (Matias et al. 2013; Vieira et al. 2022).

Along the Italian coast, there are numerous bivalve mollusk farming sites, mainly located in the north Adriatic coast, the coast of Apulia, the gulf of Liguria, and Napoli, and the lagoons of Sicily and Sardinia (Giacobbe et al. 2025). Three edible clam species, *Cerastoderma glaucum* (Bruguière, 1789), *Rudpidates decussatus* (Linnaeus, 1758) and *Polititapes aureus* (Gmelin, 1791) are indigenous to Lake Ganzirri, in the Sicilian lagoon of Capo Peloro, where they have been farmed since ancient times. Nowadays, these products are farmed by a local cooperative, marketed, consumed, and highly appreciated by the local population for their excellent taste.

*C. glaucum* is one of the most exploited bivalve species in Europe. It belongs to Cardidaee family, and it is known as “heart of the lagoon” because its heart‐like shape, very robust with evident ribs. The shell, reaching about 3 cm, has a yellow‐white‐brown color and the inside is white and orange (Santos et al. 2022).

*R. decussatus*, commonly known as the “veracious clam”, belongs to the family Veneridae and is native to Europe's coastal waters. It is commercialized over 3 cm in length, reaching a maximum size of 7 cm. Its shell is less robust than that of *C. glaucum* , with numerous tiny ribs. The shell is gray‐white in color, and the inside is white and yellow. It is the only indigenous veracious clam in the Mediterranean Sea, but it is often replaced by the Indo‐Pacific “Manila clam”, *R. philippinarum*, introduced in the Adriatic Sea for commercial reasons, but considered of lower economic value (Mamede et al. 2022).

*P. aureus* is another member of the Veneridae family, less widespread than its relative, *R. decussatus*. It is characterized by an oval shell of about 3 cm, brightly and colorfully colored, with dark brown spots or broken zigzag lines, while inside it is golden yellow, hence its specific name (Derbali and Jarboui 2021).

The aim of this study was to evaluate the fatty acids profile of the three above‐mentioned native clam species of Capo Peloro lagoon and to compare the results with samples of the same three species from the pristine Oliveri‐Tindari lagoon reserve, to assess the nutritional value of this traditional food. Moreover, commercial *R. decussatus* from the lagoons of Santa Gilla and Santa Giusta, located in Sardinia, were analyzed to compare the locally consumed Sicilian “veracious clam” with a heavily marketed analogous product.

## 2 Materials and Methods

### 2.1 Sites of Sampling

The lagoon of Capo Peloro and Oliveri‐Tindari as well as the lagoons of Santa Gilla and Santa Giusta are transitional water zones, TWZs, that indicate a peculiar ecosystem that develops at the interface between freshwater and marine environments. These zones are characterized by dynamic nature and high biodiversity and productivity (Schubert et al. 2010). Moreover, many TWZs are used for activities such as aquaculture, fishing, and tourism, but they are also vulnerable to pollution and habitat loss (Pérez‐Ruzafa et al. 2011).

The Capo Peloro lagoon is located at the northern opening of the Strait of Messina. It consists of two connected ponds, Lake Ganzirri and Lake Faro, and some canals. The lagoon is highly urbanized and hosts traditional practices of shellfish farming. This practice, of very high socio‐cultural value because its deep historical roots, has allowed to obtain the title of “heritage of ethno‐anthropological interest” and the regime of “oriented reserve” (Sanfilippo et al. 2022). Recent investigations on the trace metal content in the endemic clams cultivated in the lagoon proved absence of contamination (Giacobbe et al. 2025). A previous study carried out on sediment and clams reporting contamination by trace elements (Cullotta et al. 2008), in our opinion, is unreliable, since at least one of the studied species, namely *Chamelea gallina* (Linnaeus, 1758), is not present in the Peloro lagoon.

The Oliveri‐Tindari lagoon is a pristine environment, located on the eastern Tyrrhenian coast and subject to a protection regime; therefore, it can be considered undisturbed, except for seasonal tourism in summer (Agostino et al. 2024).

The lagoons of Santa Gilla and Santa Giusta are subject to intensive aquaculture and are located in heavily urbanized and industrial areas (Gravina et al. 2020).

### 2.2 Sample Collection

The samples collection was carried out in the cited lagoons of Sicily and Sardinia (Italy) during the late winter of both 2023 and 2024, as shown in Table S1. Annually, 6 samples of approximately 500 g for each species, *R. decussatus*, *C. glaucum* , and *P. aureus*, of commercial size, were collected by the clam farmers, under our supervision, from Lake Ganzirri in Capo Peloro lagoon, for a total of 36 samples.

Such sampling was replicated in the same period in the pristine Oliveri‐Tindari lagoon. Furthermore, commercial *R. decussatus* samples were collected during the same period from farmed clam beds in Sardinia, specifically from the lagoons of Santa Gilla (Cagliari) and Santa Giusta (Oristano).

The samples were directly transported to the laboratory, where they were kept refrigerated at −20°C until they were prepared for chemical analysis. Each group was handled independently. Clams were shucked manually by cutting the adductor muscle with a knife. Once the mollusks were separated from their shells, they were freeze‐dried using a CHRIST freeze‐dryer (model D‐37520 Osterode am Harz, Germany), then homogenized and kept in the dryer until further analysis.

### 2.3 Chemicals and Reagents

Solvents and reagents such as chloroform (≥ 99.8%), methanol (≥ 99.8%), n‐hexane (≥ 98%) and sulfuric acid (95%–97%) were obtained from Merck (Darmstadt, Germany). Certified reference solutions of fatty acid methyl esters (FAMEs, C4–C24), cod liver oil fatty acid methyl esters, and butylated hydroxytoluene (BHT) were purchased from Sigma‐Aldric (Darmstadt, Germany).

### 2.4 Fatty Acids Profile Analysis

Lipids were extracted using a chloroform‐methanol mixture according to the Folch method, with some modifications (Nava et al. 2023). Briefly, approximately 4 g of dried clams were weighed into 50 mL centrifuge tubes and mixed with 40 mL of Folch's solution (chloroform: methanol, 2:1, v/v). After stirring the mixture, approximately 5 mL of a 0.73% NaCl solution was added. The samples were then vortexed and centrifuged at 8000 rpm for 15 min at 4°C. The lipid layer was collected into a pre‐weighed flask and the solvent was evaporated using a rotary evaporator (Heidolph Instruments GmbH & Co., Schwabach, Germany). Total lipid content was measured gravimetrically. Fatty acid methyl esters (FAMEs) were subsequently prepared by hot esterification using a methanol:sulfuric acid solution (9:1, v/v) in an oven at 100°C. BHT was also added as an antioxidant to prevent PUFAs degradation by free radicals generated by heat. After 1 h, the supernatant was collected and diluted 1:2 with hexane for the analysis. The evaluation of lipid profile and the identification of each fatty acid were performed using a gas chromatograph (GC) coupled with a flame ionization detector (FID) (Dani Master GC, Dani Instrument, Milan, Italy). All analyses were conducted three times. The GC‐FID conditions are reported in Table S2.

### 2.5 Nutritional Quality of Lipid

To evaluate the nutritional quality of the FAs of clams and the possible effects on human health, several indices were evaluated.

The atherogenicity (AI) and thrombogenicity (TI) indices were determined using the following equations (Nava et al. 2023).

(1) $$\mathrm{AI}=\left[C12:0+\left(4\times C14:0\right)+C16:0\right]/\left[\Sigma \;\text{MUFA}+\Sigma \;n6\;\text{PUFA}+\Sigma \;n3\;\text{PUFA}\right]$$

(2) $$\begin{array}{cc} \mathrm{TI}\;=\; & \left(C14:0+C16:0+C18:0\right)/(0.5\times \Sigma \;\text{MUFA}+0.5\times \Sigma \;n6\;\text{PUFA}\\ & +3\times \Sigma \;n3\;\text{PUFA}+n3\;\text{PUFA}/n6\;\text{PUFA}) \end{array}$$

The AI assesses the potential of fat to promote atherosclerosis in the arteries while the TI assesses the potential to promote the formation of blood clots. The formation of thrombi and atherosclerotic plaques represents the primary cause of cardiovascular diseases such as heart attacks and strokes (Fonseca et al. 2022). High value of these indices is associated with an increased cardiovascular risk.

Another important index, the hypocholesterolaemic/hypercholesterolaemic ratio, calculated through the equation (3), evaluates the potential impacts of dietary lipids on blood cholesterol levels (Kumar Sethukali and Darshaka Jayasena 2022).

(3) $$\begin{array}{cc} h/H\;=\; & (C18:1n9+C18:2n6+C18:3n3+C20:4n6++C20:5n3\\ & +C22:5n3+C22:6n3)/\left(C14:0+C16:0\right) \end{array}$$

A higher h/H ratio suggests that the lipid composition has ipocholesterolemic effects, promoting heart health.

Moreover, the ratio of n6/n3 and the sum of EPA and DHA was evaluated.

### 2.6 Statistical Analysis

Experimental data are reported as mean ± standard deviation from triplicate analyse.

An initial multivariate matrix was constructed including all samples analyzed: *R. decussatus* from Santa Giusta and Santa Gilla lagoons, *R. decussatus*, *C. glaucum* , and *P. aureus* from Lake Ganzirri in the Capo Peloro lagoon and Oliveri‐Tindari lagoon for both 2023 and 2024, for a total of 96 cases. The matrix incorporated the variables obtained from the experimental data. Statistically significant differences in FA composition over *R. decussatus* from Sicilian and Sardinian TWZs and over different species from the same site were evaluated by Kruskal‐Wallis one‐way analysis of variance (ANOVA), followed by a post hoc Tukey's honestly significant difference (HSD). The data comparison of two different years and the same species ( *C. glaucum* and *P. aureus* ) from two Sicilian TWZs was performed through Mann–Whitney *U* test. For every variable investigated, statistical significance was accepted at the level of *p* < 0.05.

A Principal Component Analysis (PCA) was used to investigate the differentiation of clam samples according to location and year of sampling. Reducing data dimensionality highlighted differences between clam samples from various TWZs and years, allowing their representation in a dimensionless space. Only the variables that showed a significant difference were used to perform the PCA. The new data sets were first standardized to achieve uniform significance and then checked by Kaiser–Meyer–Olkin (KMO) and Bartlett tests for suitability for factor analysis. The software SPSS 13.0 for Windows (SPSS Inc., Chicago, IL, USA) was used to perform statistical analysis.

## 3 Results and Discussion

Tables 1 and 2 showed the fatty acids composition of *R. decussatus* collected in 2023 and 2024, respectively, while the Tables 3 and 4 showed the fatty acids composition of both *C. glaucum and P. aureus* collected in 2023 and 2024, respectively.

**TABLE 1:** Total fat (g/100 g ww) and fatty acids profile (% of total FA) of *R. decussatus* from different lagoons collected in 2023.

2023	*R. decussatus*			
	Capo Peloro (Lake Ganzirri)	Oliveri‐Tindari	Santa Giusta	Santa Gilla
Total fat	1.10 ± 0.10 (a)	1.06 ± 0.10 (a)	1.31 ± 0.12 (b)	1.44 ± 0.15 (b)
C12:0	0.06 ± 0.01 (a)	0.06 ± 0.01 (a)	0.09 ± 0.01 (b)	0.07 ± 0.01 (a,b)
C14:0	2.07 ± 0.19 (a,b)	1.84 ± 0.14 (a)	1.68 ± 0.17 (a)	2.49 ± 0.29 (b)
C14:1 n5	0.18 ± 0.02 (a)	0.17 ± 0.02 (a)	0.13 ± 0.01 (a)	0.54 ± 0.06 (b)
C15:0	0.81 ± 0.10 (a)	0.46 ± 0.05 (b)	0.71 ± 0.07 (a)	0.62 ± 0.06 (a,b)
C16:0	25.49 ± 2.06	22.66 ± 1.62	25.75 ± 2.57	23.79 ± 2.55
C16:1 n9	0.30 ± 0.03 (b)	0.16 ± 0.01 (a)	0.18 ± 0.02 (a)	0.20 ± 0.02 (a)
C16:1 n7	3.23 ± 0.34 (a)	3.04 ± 0.21 (a)	5.16 ± 0.42 (b)	4.80 ± 0.43 (b)
C16:1 n5	0.18 ± 0.02	0.17 ± 0.02	0.15 ± 0.02	0.16 ± 0.02
C16:2 n4	4.01 ± 0.39 (b)	2.92 ± 0.26 (a,b)	1.97 ± 0.22 (a)	1.87 ± 0.17 (a)
C16:3 n4	1.46 ± 0.12 (b)	1.13 ± 0.11 (a,b)	0.70 ± 0.09 (a)	0.65 ± 0.08 (a)
C17:0	4.08 ± 0.40 (b)	2.19 ± 0.15 (a)	1.75 ± 0.18 (a)	1.59 ± 0.14 (a)
C16:4 n4	0.64 ± 0.07 (a)	0.22 ± 0.03 (a)	2.13 ± 0.19 (b)	3.93 ± 0.30 (c)
C18:0	12.03 ± 0.71 (b)	12.11 ± 1.06 (b)	9.19 ± 0.67 (a)	8.71 ± 0.56 (a)
C18:1 n9	5.17 ± 0.39 (a,b)	5.66 ± 0.37 (b)	6.15 ± 0.40 (b)	4.24 ± 0.35 (a)
C18:1 n7	2.95 ± 0.30 (b)	2.02 ± 0.17 (a)	3.05 ± 0.27 (b)	3.30 ± 0.29 (b)
C18:2 n6	0.44 ± 0.04 (a)	0.84 ± 0.09 (b,c)	1.38 ± 0.12 (c)	0.62 ± 0.06 (b)
C18:3 n6	0.10 ± 0.01 (a)	0.10 ± 0.01 (a)	0.12 ± 0.01 (a,b)	0.14 ± 0.01 (b)
C18:3 n3	1.32 ± 0.14	1.41 ± 0.20	1.38 ± 0.13	1.43 ± 0.23
C18:4 n3	0.34 ± 0.05 (a)	0.69 ± 0.08 (b)	0.40 ± 0.04 (a)	0.52 ± 0.05 (b)
C18:4 n1	0.07 ± 0.01 (a)	0.10 ± 0.01 (a)	0.11 ± 0.01 (a)	0.34 ± 0.03 (b)
C20:0	0.73 ± 0.09 (b)	0.26 ± 0.03 (a)	0.30 ± 0.03 (a)	0.34 ± 0.03 (a)
C20:1 n11	5.55 ± 0.60 (b)	5.73 ± 0.43 (b)	4.43 ± 0.37 (a)	4.14 ± 0.36 (a)
C20:1 n9	1.28 ± 0.13 (a)	1.60 ± 0.11 (b)	1.26 ± 0.11 (a)	1.20 ± 0.10 (a)
C20:1 n7	2.13 ± 0.18 (b)	1.60 ± 0.13 (a)	2.01 ± 0.21 (b)	1.94 ± 0.18 (b)
C20:2 n6	1.64 ± 0.16 (a,b)	1.89 ± 0.15 (b)	1.30 ± 0.20 (a)	1.47 ± 0.13 (a)
C20:3 n6	0.27 ± 0.04 (b)	0.09 ± 0.01 (a)	0.31 ± 0.03 (b)	0.37 ± 0.05 (b)
C21:0	0.15 ± 0.02 (b)	0.10 ± 0.01 (a,b)	0.09 ± 0.01 (a)	0.09 ± 0.01 (a)
C20:4 n6	3.98 ± 0.31 (b)	3.30 ± 0.26 (a,b)	2.82 ± 0.23 (a)	3.02 ± 0.26 (a)
C20:3 n3	0.10 ± 0.01 (a)	0.15 ± 0.01 (a,b)	0.17 ± 0.02 (b)	0.18 ± 0.02 (b)
C20:4 n3	0.22 ± 0.03 (a,b)	0.17 ± 0.02 (a)	0.33 ± 0.03 (b,c)	0.41 ± 0.05 (c)
C20:5 n3	2.75 ± 0.31 (a)	7.46 ± 0.51 (b)	7.19 ± 0.56 (b)	8.94 ± 0.67 (b)
C22:0	0.59 ± 0.06 (c)	0.24 ± 0.03 (b)	0.03 ± 0.01 (a)	0.08 ± 0.01 (a)
C22:1 n11	0.20 ± 0.02 (a)	0.75 ± 0.08 (b)	0.27 ± 0.03 (a)	0.20 ± 0.02 (a)
C22:1 n9	0.20 ± 0.02 (b)	0.24 ± 0.02 (b)	0.13 ± 0.01 (a)	0.16 ± 0.02 (a)
C22:1 n7	0.03 ± 0.01 (b)	0.02 ± 0.01 (a,b)	0.01 ± 0.01 (a)	0.03 ± 0.00 (b)
C21:5 n3	0.76 ± 0.10 (a)	1.80 ± 0.14 (b)	1.19 ± 0.10 (a,b)	1.24 ± 0.12 (a,b)
C23:0	0.07 ± 0.01 (b)	0.01 ± 0.01 (a)	0.03 ± 0.01 (a)	0.03 ± 0.01 (a)
C22:4 n6	1.11 ± 0.10	0.99 ± 0.08	1.01 ± 0.09	1.04 ± 0.10
C22:5 n6	1.81 ± 0.17 (b)	1.06 ± 0.09 (a)	1.17 ± 0.11 (a)	1.21 ± 0.12 (a)
C22:5 n3	1.74 ± 0.15 (a,b)	1.37 ± 0.14 (a)	1.94 ± 0.18 (b)	2.02 ± 0.20 (b)
C24:0	0.22 ± 0.02 (b)	0.01 ± 0.01 (a)	0.02 ± 0.01 (a)	0.01 ± 0.00 (a)
C22:6 n3	9.59 ± 0.87 (a)	13.12 ± 0.95 (b)	11.68 ± 1.28 (a,b)	11.81 ± 1.09 (a,b)
C24:1 n9	0.01 ± 0.01 (a)	0.10 ± 0.01 (b,c)	0.19 ± 0.02 (c)	0.07 ± 0.01 (a,b)
SFAs	46.29 ± 1.90 (b)	39.95 ± 2.31 (a)	39.62 ± 2.75 (a,b)	37.81 ± 2.56 (a)
MUFAs	21.39 ± 1.61	21.25 ± 1.38	23.12 ± 1.52	20.98 ± 1.45
PUFAs	32.32 ± 0.99 (a)	38.80 ± 1.49 (b)	37.26 ± 1.54 (a,b)	41.21 ± 1.57 (b)
∑ n6	9.34 ± 0.28 (b)	8.26 ± 0.64 (a,b)	8.09 ± 0.62 (a)	7.88 ± 0.60 (a)
∑ n3	16.82 ± 0.94 (a)	26.17 ± 0.95 (b)	24.27 ± 1.31 (b)	26.55 ± 1.49 (b)
n6:n3 ratio	0.56 ± 0.03 (b)	0.32 ± 0.03 (a)	0.33 ± 0.04 (a)	0.30 ± 0.03 (a)
AI	0.71 ± 0.05 (b)	0.54 ± 0.04 (a)	0.59 ± 0.06 (a,b)	0.61 ± 0.05 (a,b)
TI	0.59 ± 0.05 (b)	0.38 ± 0.03 (a)	0.40 ± 0.05 (a,b)	0.36 ± 0.04 (a)
h/H	0.91 ± 0.07 (a)	1.36 ± 0.11 (b)	1.20 ± 0.14 (a,b)	1.23 ± 0.13 (b)
EPA + DHA	12.34 ± 0.77 (a)	20.59 ± 0.89 (b)	18.87 ± 1.19 (b)	20.75 ± 1.45 (b)

*Note:* Values are expressed as mean ± SD of *n* = 6 per group, each analyzed in triplicate. Different letters in the same raw represent statistically different results (*p* < 0.05) obtained by Kruskal–Wallis test for clams collected in different lagoons.

**TABLE 2:** Total fat (g/100 g ww) and fatty acids profile (% of total FA) of *R. decussatus* from different lagoons collected in 2024.

2024	*R. decussatus*			
	Capo Peloro (Lake Ganzirri)	Oliveri‐Tindari	Santa Giusta	Santa Gilla
Total fat	1.00 ± 0.10 (a)	0.96 ± 0.09 (a)	1.18 ± 0.11 (a,b)	1.31 ± 0.12 (b)
C12:0	0.46 ± 0.05 (b)	0.09 ± 0.02 (a)	0.33 ± 0.04 (b)	0.08 ± 0.01 (a)
C14:0	4.14 ± 0.28 (c)	3.27 ± 0.51 (b)	2.45 ± 0.22 (a,b)	1.78 ± 0.17 (a)
C14:1 n5	0.22 ± 0.02 (b)	0.14 ± 0.07 (b)	0.05 ± 0.01 (a)	0.08 ± 0.01 (a,b)
C15:0	0.99 ± 0.06 (b)	0.42 ± 0.08 (a)	0.91 ± 0.11 (b)	0.88 ± 0.09 (b)
C16:0	28.58 ± 1.63 (a,b)	31.79 ± 2.88 (b)	25.21 ± 2.48 (a)	25.04 ± 2.07 (a)
C16:1 n9	0.95 ± 0.07 (b)	0.41 ± 0.07 (a)	0.41 ± 0.04 (a)	0.44 ± 0.05 (a)
C16:1 n7	3.30 ± 0.21 (a)	3.88 ± 0.30 (a,b)	4.12 ± 0.40 (b)	4.07 ± 0.38 (b)
C16:1 n5	0.23 ± 0.05 (b)	0.41 ± 0.04 (c)	0.14 ± 0.01 (a)	0.41 ± 0.05 (c)
C16:2 n4	3.68 ± 0.25 (b)	1.21 ± 0.41 (a)	1.63 ± 0.17 (a)	1.97 ± 0.18 (a,b)
C16:3 n4	0.42 ± 0.03 (a)	0.63 ± 0.22 (a,b)	0.89 ± 0.09 (b)	1.03 ± 0.13 (b)
C17:0	4.57 ± 0.48 (b)	1.70 ± 0.44 (a)	1.63 ± 0.16 (a)	1.96 ± 0.21 (a)
C16:4 n4	0.41 ± 0.03 (b)	0.08 ± 0.01 (a)	0.15 ± 0.01 (a)	0.18 ± 0.02 (a)
C18:0	10.93 ± 1.07 (b)	7.99 ± 0.64 (a)	8.41 ± 0.86 (a)	9.02 ± 0.99 (a,b)
C18:1 n9	5.91 ± 0.39 (b)	5.04 ± 0.78 (a,b)	4.60 ± 0.56 (a,b)	3.66 ± 0.43 (a)
C18:1 n7	1.95 ± 0.23 (a)	1.90 ± 0.46 (a)	3.52 ± 0.51 (b)	3.06 ± 0.30 (b)
C18:2 n6	0.51 ± 0.11 (a)	0.77 ± 0.08 (a,b)	0.88 ± 0.08 (b)	0.73 ± 0.10 (a,b)
C18:3 n6	0.11 ± 0.01	0.15 ± 0.03	0.13 ± 0.01	0.13 ± 0.01
C18:3 n3	0.76 ± 0.05 (a)	0.92 ± 0.15 (a,b)	1.31 ± 0.14 (b)	1.31 ± 0.15 (b)
C18:4 n3	1.40 ± 0.13 (a)	2.00 ± 0.22 (a,b)	2.22 ± 0.21 (b)	2.29 ± 0.22 (b)
C18:4 n1	0.16 ± 0.01 (b)	0.07 ± 0.02 (a)	0.05 ± 0.01 (a)	0.06 ± 0.01 (a)
C20:0	0.22 ± 0.01 (a)	0.23 ± 0.14 (a,b)	0.32 ± 0.05 (a,b)	0.41 ± 0.10 (b)
C20:1 n11	1.61 ± 0.19 (a)	1.80 ± 0.24 (a)	2.14 ± 0.22 (a,b)	2.57 ± 0.34 (b)
C20:1 n9	0.76 ± 0.15 (a)	2.27 ± 0.20 (b)	1.62 ± 0.14 (a,b)	2.08 ± 0.24 (b)
C20:1 n7	0.50 ± 0.07 (a)	1.12 ± 0.18 (a,b)	1.29 ± 0.12 (b)	1.59 ± 0.19 (b)
C20:2 n6	0.70 ± 0.09 (a)	1.43 ± 0.33 (a,b)	1.63 ± 0.28 (b)	1.84 ± 0.19 (b)
C20:3 n6	0.06 ± 0.01 (a)	0.14 ± 0.06 (a,b)	0.24 ± 0.03 (b)	0.19 ± 0.02 (b)
C21:0	0.20 ± 0.04 (b)	0.13 ± 0.02 (a)	0.14 ± 0.02 (a)	0.14 ± 0.01 (a)
C20:4 n6	2.83 ± 0.43 (b)	2.05 ± 0.25 (a)	2.46 ± 0.25 (a,b)	2.52 ± 0.26 (a,b)
C20:3 n3	0.07 ± 0.01 (a)	0.24 ± 0.12 (a,b)	0.45 ± 0.05 (b)	0.35 ± 0.03 (a,b)
C20:4 n3	0.21 ± 0.04 (a)	0.50 ± 0.16 (a,b)	0.88 ± 0.09 (b)	0.81 ± 0.12 (b)
C20:5 n3	7.35 ± 0.71 (a)	7.54 ± 0.71 (a)	8.34 ± 0.77 (a,b)	9.50 ± 0.87 (b)
C22:0	0.69 ± 0.15 (b)	0.53 ± 0.14 (a,b)	0.44 ± 0.05 (a)	0.48 ± 0.06 (a)
C22:1 n11	0.14 ± 0.01 (a)	0.18 ± 0.01 (b)	0.17 ± 0.01 (a,b)	0.18 ± 0.01 (b)
C22:1 n9	0.12 ± 0.01 (a)	0.22 ± 0.06 (b)	0.18 ± 0.02 (b)	0.16 ± 0.01 (a,b)
C22:1 n7	0.14 ± 0.01 (a)	0.18 ± 0.09 (a,b)	0.14 ± 0.01 (a)	0.24 ± 0.03 (b)
C21:5 n3	1.74 ± 0.19 (b)	1.05 ± 0.10 (a)	1.56 ± 0.32 (b)	1.33 ± 0.13 (a,b)
C23:0	0.05 ± 0.01	0.05 ± 0.02	0.06 ± 0.01	0.07 ± 0.01
C22:4 n6	0.64 ± 0.04 (a)	0.81 ± 0.16 (a,b)	0.84 ± 0.09 (b)	0.88 ± 0.11 (b)
C22:5 n6	0.84 ± 0.10 (a)	1.10 ± 0.11 (b)	0.99 ± 0.12 (a,b)	1.10 ± 0.13 (b)
C22:5 n3	1.11 ± 0.08 (a)	1.46 ± 0.17 (a,b)	2.74 ± 0.30 (c)	1.79 ± 0.19 (b,c)
C24:0	0.05 ± 0.01 (a)	0.16 ± 0.05 (a,b)	0.19 ± 0.03 (b)	0.21 ± 0.02 (b)
C22:6 n3	10.20 ± 1.11 (a)	13.48 ± 1.22 (b)	13.17 ± 1.65 (b)	12.84 ± 1.17 (a,b)
C24:1 n9	0.10 ± 0.01 (a)	0.49 ± 0.15 (a,b)	1.01 ± 0.11 (b)	0.59 ± 0.06 (a,b)
SFAs	50.88 ± 1.69 (b)	46.35 ± 1.92 (a,b)	40.08 ± 1.85 (a)	40.07 ± 0.77 (a)
MUFAs	15.92 ± 0.42 (a)	18.04 ± 1.59 (a,b)	19.38 ± 1.82 (b)	19.11 ± 1.61 (b)
PUFAs	33.20 ± 1.47 (a)	35.61 ± 1.94 (a,b)	40.55 ± 1.26 (b)	40.83 ± 1.05 (b)
∑ n6	5.70 ± 0.33 (a)	6.44 ± 0.60 (a,b)	7.16 ± 0.51 (b)	7.39 ± 0.46 (b)
∑ n3	22.85 ± 1.50 (a)	27.18 ± 1.29 (a,b)	30.67 ± 1.92 (b)	30.20 ± 1.49 (b)
n6:n3 ratio	0.25 ± 0.02	0.24 ± 0.03	0.24 ± 0.03	0.25 ± 0.02
AI	1.03 ± 0.06 (b)	0.87 ± 0.09 (a,b)	0.62 ± 0.05 (a)	0.57 ± 0.05 (a)
TI	0.53 ± 0.05 (b)	0.44 ± 0.03 (a,b)	0.33 ± 0.11 (a)	0.33 ± 0.01 (a)
h/H	0.88 ± 0.09 (a)	0.90 ± 0.06 (a)	1.22 ± 0.11 (b)	1.21 ± 0.05 (b)
EPA + DHA	17.56 ± 1.56 (a)	21.02 ± 1.29 (b)	21.51 ± 2.39 (b)	22.34 ± 2.01 (b)

*Note:* Values are expressed as mean ± SD of *n* = 6 per group, each analyzed in triplicate. Different letters in the same raw represent statistically different results (*p* < 0.05) obtained by Kruskal–Wallis test for clams collected in different lagoons.

**TABLE 3:** Total fat (g/100 g ww) and fatty acids profile (% of total FA) of *C. glaucum* and *P. aureus* from Sicilian lagoons collected in 2023.

2023	*C. glaucum*			*P. aureus*		
	Capo Peloro (Lake Ganzirri)	Oliveri‐Tindari	*p* value*	Capo Peloro (Lake Ganzirri)	Oliveri‐Tindari	*p* value*
Total fat	1.01 ± 0.09	0.97 ± 0.09	0.589	1.05 ± 0.10	1.03 ± 0.09	0.699
C12:0	0.14 ± 0.02	0.13 ± 0.04	0.699	0.04 ± 0.01	0.06 ± 0.01	< 0.05
C14:0	3.54 ± 0.29	1.61 ± 0.18	< 0.05	4.19 ± 1.61	2.93 ± 0.29	0.394
C14:1 n5	0.28 ± 0.03	0.30 ± 0.03	0.310	0.22 ± 0.02	0.27 ± 0.03	< 0.05
C15:0	1.50 ± 0.14	1.08 ± 0.09	< 0.05	0.85 ± 0.16	1.13 ± 0.12	< 0.05
C16:0	26.35 ± 1.86	22.37 ± 1.96	< 0.05	25.74 ± 1.89	24.73 ± 2.44	0.589
C16:1 n9	0.19 ± 0.01	0.29 ± 0.03	< 0.05	0.32 ± 0.09	0.30 ± 0.03	0.699
C16:1 n7	4.25 ± 0.33	3.10 ± 0.24	< 0.05	3.65 ± 0.25	3.12 ± 0.28	< 0.05
C16:1 n5	0.23 ± 0.02	0.09 ± 0.02	< 0.05	0.18 ± 0.02	0.20 ± 0.02	0.240
C16:2 n4	2.68 ± 0.22	2.97 ± 0.30	0.093	3.06 ± 0.31	2.70 ± 0.26	< 0.05
C16:3 n4	1.14 ± 0.12	1.10 ± 0.11	0.818	1.84 ± 0.13	1.12 ± 0.12	< 0.05
C17:0	3.34 ± 0.31	2.54 ± 0.18	< 0.05	2.88 ± 0.27	2.78 ± 0.30	0.598
C16:4 n4	0.71 ± 0.11	1.65 ± 0.19	< 0.05	0.69 ± 0.18	0.67 ± 0.07	0.818
C18:0	10.88 ± 0.84	11.41 ± 1.14	0.485	8.47 ± 0.74	10.50 ± 1.00	< 0.05
C18:1 n9	2.82 ± 0.26	3.41 ± 0.25	< 0.05	4.49 ± 0.30	4.17 ± 0.30	0.132
C18:1 n7	2.61 ± 0.18	2.14 ± 0.18	< 0.05	3.48 ± 0.78	2.64 ± 0.30	< 0.05
C18:2 n6	0.80 ± 0.07	0.99 ± 0.10	< 0.05	1.08 ± 0.44	0.79 ± 0.09	0.699
C18:3 n6	0.16 ± 0.02	0.26 ± 0.03	< 0.05	0.11 ± 0.01	0.12 ± 0.01	0.065
C18:3 n3	1.31 ± 0.11	1.16 ± 0.10	< 0.05	1.23 ± 0.27	1.28 ± 0.13	1.000
C18:4 n3	0.23 ± 0.03	1.19 ± 0.11	< 0.05	1.53 ± 0.18	0.44 ± 0.05	< 0.05
C18:4 n1	0.12 ± 0.01	0.01 ± 0.00	< 0.05	0.02 ± 0.02	0.01 ± 0.00	0.485
C20:0	0.33 ± 0.03	0.33 ± 0.03	1.000	0.37 ± 0.04	0.46 ± 0.05	< 0.05
C20:1 n11	5.45 ± 0.50	5.76 ± 0.50	0.310	3.78 ± 0.30	4.23 ± 0.34	< 0.05
C20:1 n9	1.87 ± 0.13	0.99 ± 0.07	< 0.05	1.05 ± 0.14	0.99 ± 0.12	0.485
C20:1 n7	2.05 ± 0.13	2.14 ± 0.19	0.485	1.59 ± 0.20	1.83 ± 0.17	< 0.05
C20:2 n6	1.22 ± 0.13	1.36 ± 0.17	0.132	1.62 ± 0.21	1.57 ± 0.16	0.818
C20:3 n6	0.28 ± 0.04	0.39 ± 0.04	< 0.05	0.27 ± 0.05	0.36 ± 0.04	< 0.05
C21:0	0.06 ± 0.01	0.19 ± 0.02	< 0.05	0.12 ± 0.05	0.19 ± 0.02	< 0.05
C20:4 n6	4.63 ± 0.33	3.31 ± 0.31	< 0.05	2.69 ± 0.38	2.55 ± 0.29	0.485
C20:3 n3	0.21 ± 0.02	0.20 ± 0.02	0.394	0.19 ± 0.13	0.08 ± 0.01	0.485
C20:4 n3	0.11 ± 0.01	0.47 ± 0.05	< 0.05	0.31 ± 0.17	0.20 ± 0.02	0.937
C20:5 n3	5.30 ± 0.50	7.78 ± 0.72	< 0.05	5.54 ± 0.47	6.37 ± 0.60	< 0.05
C22:0	0.09 ± 0.01	0.08 ± 0.01	< 0.05	0.05 ± 0.01	0.05 ± 0.01	0.818
C22:1 n11	0.19 ± 0.02	0.93 ± 0.10	< 0.05	0.39 ± 0.05	0.42 ± 0.05	0.310
C22:1 n9	0.15 ± 0.01	0.61 ± 0.07	< 0.05	0.18 ± 0.02	0.22 ± 0.03	< 0.05
C22:1 n7	0.04 ± 0.01	0.07 ± 0.01	< 0.05	0.19 ± 0.20	0.16 ± 0.02	0.180
C21:5 n3	1.34 ± 0.13	1.37 ± 0.12	0.589	0.89 ± 0.44	0.97 ± 0.09	1.000
C23:0	0.02 ± 0.01	0.08 ± 0.01	< 0.05	0.05 ± 0.02	0.09 ± 0.01	< 0.05
C22:4 n6	0.99 ± 0.10	1.27 ± 0.09	< 0.05	1.21 ± 0.09	1.41 ± 0.14	< 0.05
C22:5 n6	1.13 ± 0.13	1.50 ± 0.13	< 0.05	1.49 ± 0.21	1.97 ± 0.16	< 0.05
C22:5 n3	1.31 ± 0.11	1.56 ± 0.14	< 0.05	1.48 ± 0.14	1.64 ± 0.16	0.180
C24:0	0.03 ± 0.01	0.02 ± 0.01	0.310	0.02 ± 0.01	0.02 ± 0.01	1.000
C22:6 n3	9.84 ± 1.02	11.74 ± 1.23	< 0.05	12.37 ± 1.28	14.09 ± 1.36	0.093
C24:1 n9	0.15 ± 0.01	0.10 ± 0.01	< 0.05	0.11 ± 0.06	0.20 ± 0.02	< 0.05
SFAs	46.26 ± 1.96	39.83 ± 2.76	< 0.05	42.78 ± 1.19	42.92 ± 2.80	1.000
MUFAs	20.25 ± 1.27	19.91 ± 1.51	0.699	19.62 ± 1.46	18.74 ± 1.51	0.394
PUFAs	33.49 ± 0.90	40.26 ± 1.87	< 0.05	37.60 ± 1.24	38.34 ± 1.68	0.394
∑ n6	9.21 ± 0.39	9.07 ± 0.75	0.818	8.46 ± 0.35	8.78 ± 0.79	0.394
∑ n3	19.64 ± 1.08	25.47 ± 1.21	< 0.05	23.53 ± 1.06	25.07 ± 1.02	0.093
n6:n3 ratio	0.47 ± 0.04	0.36 ± 0.03	< 0.05	0.36 ± 0.01	0.35 ± 0.03	0.589
AI	0.83 ± 0.05	0.53 ± 0.06	< 0.05	0.83 ± 0.12	0.70 ± 0.07	0.093
TI	0.54 ± 0.05	0.38 ± 0.04	< 0.05	0.44 ± 0.03	0.42 ± 0.04	0.394
h/H	0.87 ± 0.08	1.26 ± 0.14	< 0.05	0.97 ± 0.07	1.12 ± 0.12	< 0.05
EPA + DHA	15.13 ± 0.86	19.53 ± 0.99	< 0.05	17.91 ± 1.14	20.45 ± 0.98	< 0.05

*Note:* Values are expressed as mean ± SD of *n* = 6 per group, each analyzed in triplicate.

* Statistical results for clams collected in different lagoons obtained by Mann–Whitney test (*p* < 0.05).

**TABLE 4:** Total fat (g/100 g ww) and fatty acids profile (% of total FA) of *C. glaucum* and *P. aureus* from Sicilian lagoons collected in 2024.

2024	*C. glaucum*			*P. aureus*		
	Capo Peloro (Lake Ganzirri)	Oliveri‐Tindari	*p* value*	Capo Peloro (Lake Ganzirri)	Oliveri‐Tindari	*p* value*
Total fat	1.12 ± 0.08	1.07 ± 0.09	0.132	1.17 ± 0.12	1.13 ± 0.10	0.485
C12:0	0.17 ± 0.02	0.04 ± 0.01	< 0.05	0.09 ± 0.02	0.07 ± 0.01	0.180
C14:0	2.55 ± 0.18	2.79 ± 0.28	0.132	1.54 ± 0.35	2.38 ± 0.24	< 0.05
C14:1 n5	0.16 ± 0.02	0.09 ± 0.01	< 0.05	0.07 ± 0.01	0.06 ± 0.01	< 0.05
C15:0	0.85 ± 0.10	0.36 ± 0.08	< 0.05	0.55 ± 0.06	0.53 ± 0.10	0.699
C16:0	28.65 ± 2.08	29.33 ± 2.28	0.699	26.02 ± 1.82	31.88 ± 2.05	< 0.05
C16:1 n9	0.31 ± 0.06	0.47 ± 0.06	< 0.05	0.48 ± 0.04	0.11 ± 0.02	< 0.05
C16:1 n7	2.44 ± 0.19	3.98 ± 0.34	< 0.05	1.50 ± 0.11	5.32 ± 0.52	< 0.05
C16:1 n5	0.12 ± 0.04	0.12 ± 0.03	< 0.05	0.09 ± 0.01	0.53 ± 0.07	< 0.05
C16:2 n4	2.72 ± 0.26	1.17 ± 0.13	< 0.05	3.24 ± 0.24	1.77 ± 0.16	< 0.05
C16:3 n4	0.61 ± 0.06	0.36 ± 0.13	< 0.05	1.20 ± 0.10	0.69 ± 0.09	< 0.05
C17:0	3.61 ± 0.43	1.92 ± 0.59	< 0.05	3.36 ± 0.37	1.85 ± 0.24	< 0.05
C16:4 n4	0.59 ± 0.07	0.05 ± 0.01	< 0.05	0.38 ± 0.06	0.06 ± 0.02	< 0.05
C18:0	11.00 ± 1.08	9.19 ± 0.91	< 0.05	10.54 ± 0.87	9.98 ± 1.14	0.310
C18:1 n9	3.36 ± 0.32	6.98 ± 0.59	< 0.05	4.93 ± 0.49	4.59 ± 0.37	0.180
C18:1 n7	2.50 ± 0.19	4.69 ± 0.42	< 0.05	1.80 ± 0.16	2.97 ± 0.30	< 0.05
C18:2 n6	0.90 ± 0.18	1.09 ± 0.11	0.065	0.51 ± 0.09	0.32 ± 0.03	< 0.05
C18:3 n6	0.15 ± 0.05	0.05 ± 0.01	< 0.05	0.10 ± 0.02	0.06 ± 0.01	< 0.05
C18:3 n3	0.75 ± 0.08	1.17 ± 0.11	< 0.05	0.89 ± 0.09	0.36 ± 0.09	< 0.05
C18:4 n3	1.18 ± 0.11	3.72 ± 0.36	< 0.05	1.74 ± 0.18	0.82 ± 0.11	< 0.05
C18:4 n1	0.16 ± 0.03	0.10 ± 0.01	< 0.05	0.07 ± 0.01	0.41 ± 0.07	< 0.05
C20:0	0.29 ± 0.17	0.19 ± 0.10	0.180	0.18 ± 0.02	0.17 ± 0.02	0.394
C20:1 n11	2.00 ± 0.23	1.20 ± 0.15	< 0.05	1.83 ± 0.18	1.58 ± 0.12	< 0.05
C20:1 n9	2.38 ± 0.23	2.68 ± 0.25	0.132	1.76 ± 0.19	1.61 ± 0.13	0.180
C20:1 n7	1.34 ± 0.11	1.03 ± 0.13	< 0.05	1.02 ± 0.13	0.99 ± 0.12	0.485
C20:2 n6	1.27 ± 0.15	1.14 ± 0.12	0.132	1.81 ± 0.18	2.29 ± 0.19	< 0.05
C20:3 n6	0.13 ± 0.02	0.07 ± 0.02	< 0.05	0.17 ± 0.03	0.09 ± 0.01	< 0.05
C21:0	0.15 ± 0.01	0.06 ± 0.01	< 0.05	0.17 ± 0.03	0.11 ± 0.01	< 0.05
C20:4 n6	2.56 ± 0.46	1.42 ± 0.20	< 0.05	2.47 ± 0.25	1.15 ± 0.10	< 0.05
C20:3 n3	0.20 ± 0.04	0.63 ± 0.09	< 0.05	0.21 ± 0.02	0.23 ± 0.06	0.589
C20:4 n3	0.46 ± 0.10	0.78 ± 0.12	< 0.05	0.45 ± 0.06	0.50 ± 0.09	0.310
C20:5 n3	7.90 ± 0.54	5.31 ± 0.60	< 0.05	4.90 ± 0.34	4.45 ± 0.41	0.132
C22:0	0.27 ± 0.04	0.14 ± 0.02	< 0.05	0.47 ± 0.06	0.46 ± 0.06	0.818
C22:1 n11	0.48 ± 0.29	0.52 ± 0.08	1.000	0.22 ± 0.05	0.26 ± 0.04	0.132
C22:1 n9	0.19 ± 0.01	0.16 ± 0.03	0.180	0.18 ± 0.02	0.22 ± 0.04	< 0.05
C22:1 n7	0.06 ± 0.05	0.07 ± 0.01	1.000	0.03 ± 0.01	0.09 ± 0.01	< 0.05
C21:5 n3	2.42 ± 0.42	1.20 ± 0.11	< 0.05	2.01 ± 0.22	1.11 ± 0.13	< 0.05
C23:0	0.04 ± 0.01	0.03 ± 0.01	0.310	0.06 ± 0.02	0.02 ± 0.01	< 0.05
C22:4 n6	0.75 ± 0.07	0.19 ± 0.05	< 0.05	1.22 ± 0.12	0.58 ± 0.10	< 0.05
C22:5 n6	0.97 ± 0.09	0.47 ± 0.12	< 0.05	1.53 ± 0.14	0.88 ± 0.09	< 0.05
C22:5 n3	1.41 ± 0.16	1.61 ± 0.21	0.065	2.63 ± 0.23	1.45 ± 0.18	< 0.05
C24:0	0.10 ± 0.05	0.07 ± 0.01	0.485	0.09 ± 0.02	0.09 ± 0.02	0.818
C22:6 n3	11.72 ± 0.86	13.33 ± 1.31	0.065	17.48 ± 1.43	16.86 ± 1.18	0.485
C24:1 n9	0.14 ± 0.07	0.05 ± 0.01	< 0.05	0.03 ± 0.01	0.10 ± 0.02	< 0.05
SFAs	47.67 ± 2.14	44.10 ± 2.56	< 0.05	43.08 ± 2.11	47.56 ± 2.07	< 0.05
MUFAs	15.49 ± 1.20	22.04 ± 1.55	< 0.05	13.93 ± 0.94	18.39 ± 1.45	< 0.05
PUFAs	36.85 ± 2.30	33.86 ± 1.77	< 0.05	43.00 ± 1.24	34.05 ± 1.44	< 0.05
∑ n6	6.73 ± 0.66	4.43 ± 0.29	< 0.05	7.80 ± 0.57	5.35 ± 0.33	< 0.05
∑ n3	26.05 ± 1.89	27.76 ± 1.45	0.180	30.31 ± 1.05	25.78 ± 1.22	< 0.05
n6:n3 ratio	0.26 ± 0.03	0.16 ± 0.01	< 0.05	0.26 ± 0.02	0.21 ± 0.01	< 0.05
AI	0.81 ± 0.06	0.75 ± 0.06	0.132	0.62 ± 0.07	0.84 ± 0.06	< 0.05
TI	0.45 ± 0.04	0.40 ± 0.03	< 0.05	0.36 ± 0.03	0.47 ± 0.03	< 0.05
h/H	0.92 ± 0.08	0.97 ± 0.09	0.310	1.23 ± 0.10	0.85 ± 0.05	< 0.05
EPA + DHA	19.62 ± 1.37	18.64 ± 1.47	0.180	22.38 ± 1.21	21.31 ± 0.91	0.132

*Note:* Values are expressed as mean ± SD of *n* = 6 per group, each analyzed in triplicate.

* Statistical results for clams collected in different lagoons obtained by Mann–Whitney test (*p* < 0.05).

Total lipid constituted about 1% of the weight of the clams. The content of fat as well as the fatty acids profile is strictly related to the gametogenic cycle, in turn related to season, environmental conditions, and food availability. The lipid content, in fact, increases progressively according to the availability of food as an energy reserve before gametogenesis. During the colder winter months, the gonads remain inactive. As temperatures rise in the spring, gonadal development begins and clams store energy reserves for gamete production (Matias et al. 2013), thus explaining why clams are generally more appreciated between late winter and early spring, coinciding with our sampling time. The lipid content of *C. glaucum* and *P. aureus* is similar to that reported in the literature (Bityutskaya et al. 2024; Mohammad and Yusuf 2016) while contrasting data are available about *R. decussatus* (Bejaoui et al. 2020).

Forty‐three fatty acids in the lipid extracted were found with the number of carbon atoms from 12 to 24. Figure S1 shows a representative GC‐FID chromatogram of the fatty acids identified. The samples were characterized by a main content of saturated fatty acids (SFAs, 39.62%–50.88%), followed by polyunsaturated fatty acids (PUFAs, 32.32%–43.00%) and monounsaturated fatty acids (MUFAs, 13.93%–23.12%). In fact, the main fatty acid was palmitic acid (C16:0) in all samples at a concentration from 22.37% in *C. glaucum* sampled in Oliveri‐Tindari in 2023, to 31.88% in *P. aureus* from Oliveri‐Tindari collected in 2024.

Other predominant fatty acids were DHA (C22:6 n3, ranging from 9.59% in *R. decussatus* from Capo Peloro 2023 to 17.48% in *P. aureus* from Oliveri‐Tindari 2024), stearic (C18:0, from 7.99% in *R. decussatus* from Oliveri‐Tindari 2024 to 12.11% in *R. decusssatus* from Oliveri‐Tindari 2023) and EPA (C22:5 n3, from 2.75% in *R. decussatus* from Capo Peloro 2023 to 9.50% in *R. decussatus* from Santa Gilla 2024).

To the best of the authors' knowledge, the literature regarding the fatty acid profile in these clam species is limited. Only some studies have been conducted on the fatty acid profile of *R. decussatus*. The data available slightly differ from our results principally for the content of SFAs (Bejaoui et al. 2020, 2019, 2018), that are higher in our samples at the cost of PUFAs. However, the nutritional quality of lipids remains comparable to that reported by Anacleto et al. (Anacleto et al. 2014).

These differences in respect to literature data may result from the different storage conditions, sites, or seasons of sampling. Among the samples here analyzed, the fatty acids profile showed a great variability related to the different species, collection sites, and years of sampling.

Comparing the results obtained from *R. decussatus*, the Sardinian samples are characterized by a higher lipid content than Sicilian clams (*p* < 0.05) in both years (Tables 1 and 2). Focusing on samples collected in 2023 (Table 1), there was no statistical difference in the content of C16:0 (about 24%), while the content of oleic acid (C16:1 n7) was higher in samples from Sardinia (5.16% and 4.80%) than in samples from Sicily (3.23 and 3.04%). *R. decussatus* from Capo Peloro was characterized by a higher content of C17:0 (4.08%), C20:4 n6 (3.98%) and C22:5 n6 (1.81%) than the samples from other sites. On the contrary, the lowest concentration of EPA was recorded in clams from Capo Peloro (2.75%); therefore, the sum of n3 series fatty acids and of EPA and DHA was rather low (16.82% and 12.34%, respectively) compared with the samples collected in other sites (about 25% and 20%, respectively). The Sardinian clams had a lower content of stearic acid (9.19% and 8.71%) and C20:1 n11 (4.43% and 4.14%) than Sicilian clams (12.03% and 12.11%, 5.55% and 5.73%, respectively). Clams from Capo Peloro showed a higher content of SFAs and a lower content of PUFAs, but there was no statistical difference in the content of MUFAs among samples from Sicily and Sardinia. A similar trend was seen in the subsequent year (Table 2), since samples from Capo Peloro recorded the highest content of SFAs (50.88%), followed by samples from Oliveri‐Tindari (46.35%) and Sardinia (about 40%). The samples from Capo Peloro differed from the Sardinian samples for many fatty acids, while the profile of fatty acids of the Oliveri‐Tindari samples had intermediate values. The C16:0 and C18:0 followed a different trend because C16:0 content was higher in samples from Oliveri‐Tindari (31.79%), followed by Capo Peloro (28.58%) and Sardinia (about 25%), while C18:0 content was higher in samples from Capo Peloro (10.93%), followed by Santa Gilla (9.02%), Oristano (8.41%) and Oliveri‐Tindari (7.99%). The levels of EPA were low in Sicilian clams (about 7%), while they increased in Sardinian clams (8.34% and 9.50% in samples from Oristano and Santa Gilla, respectively). Both in 2023 and 2024, the highest values of DHA were registered in samples from Oliveri‐Tindari (13.12% and 13.48%, respectively), followed by Sardinian samples and Capo Peloro samples that showed the lowest content (9.59% and 10.20%, respectively).

The higher content of PUFAs in clams from Sardinia than from Sicily may be related to the difference in air temperature between the two islands. In fact, the mean temperature in Messina during January and February was higher than the mean temperature in Sardinia (Copernicus 2024). According to previous study, the degree of instauration increases with low temperature, with the purpose of maintaining the membrane fluidity compromised by the low temperature (Bejaoui et al. 2019). Another factor affecting the fatty acid profile is pollution. Transition metals (such as As, Cr, Cd Co, Cu, Hg, Pb), in fact, can stimulate the peroxidation of the membrane lipids resulting in the peroxidation of lipid radicals and in the formation of lipid degradation products (Mansour et al. 2020).

Regarding *P. aureus* collected in 2023 (Table 3), the total lipid content does not statistically differ between the two sites in Sicily. Moreover, there were no statistical differences among SFAs, MUFAs, and PUFAs, and the sum of n3 and n6 fatty acids between clams from Capo Peloro and Oliveri‐Tindari. A notable difference (*p* < 0.05) was found in the total content of EPA and DHA, which was higher in the samples from Oliveri‐Tindari (20.45%) than in those from Capo Peloro (17.91%). In 2024 (Table 4), although the lipid content remained similar, significant differences were evident in the samples collected in the Sicilian sites. Clams from Capo Peloro, in fact, had a significantly higher content of PUFAs (43.00%), n3, and n6 fatty acids (30.31% and 7.80%) and a lower content of SFAs (43.08%) and MUFAs (13.93%) than clams from Oliveri‐Tindari (*p* < 0.05). Despite the level of EPA and DHA and their sum being higher in samples from Capo Peloro, this difference was not statistically significant.

Turning on *C. glaucum* , there are no significant differences between the lipid content from Capo Peloro and Oliveri‐Tindari samples in both years (Tables 3 and 4). Moreover, samples collected in 2024 are characterized by a similar EPA and DHA sum and PUFAs n3 content, and by statistical differences in SFAs, MUFAs, and PUFAs. Samples from Capo Peloro, in fact, have a higher content of SFAs and PUFAs, including that of the n6 series, and a lower content of MUFAs than samples from Oliveri‐Tindari (Table 4). In 2023, the content of SFAs showed a significant increase in samples from Capo Peloro compared to those from Oliveri‐Tindari lagoon at the cost of PUFAs, mainly due to the reduction of the n3 series, including EPA and DHA (Table 3).

Table S3 shows the statistical comparison of the fatty acid profile of clams collected in the 2 years.

In general, all species from the lagoon of Capo Peloro collected in 2024 showed a significant higher content of PUFAs n3, among them EPA and DHA, and a significant lower content of MUFAs than sample collected in 2023. A different trend was found in the species from Oliveri‐Tindari: in 2024, there was a significant increase in SFAs in all species against a reduction in PUFAs, mainly of the n6 series. Clams from the lagoons of Sardinia, on the other hand, had a statistical increase in n3 fatty acids. This increase in the samples from Santa Gilla remained isolated and did not affect the main fatty acids (EPA and DHA). On the contrary, in the samples from Santa Giusta there was a significant increase of EPA and DHA, which led to a considerable increase of total PUFAs against a reduction of MUFAs. Considering that the sampling season was the same for all species, sites and years, exogenous factors such as temperatures and food availability might explain these differences. In fact, the Copernicus Climate Change Service confirms 2024 as the warmest year ever recorded globally and the highest sea temperatures were also reached (Copernicus 2024). Discordance between temperature increase and availability of food has been reported in literature as potential cause of depletion in growth and development of clams (Zarnoch and Schreibman 2008).

Comparing the three collected species in the Sicilian lagoons (Figure S2), the content of SFAs does not change among the species from Oliveri‐Tindari in both years. On the contrary, *P. aureus* from Capo Peloro is characterized by a lower content of SFAs and MUFAs and a higher content of PUFAs than the other species.

### 3.1 Nutritional Quality of Lipid

The clam species analyzed in this investigation, as generally known for fishes and seafood, are characterized by good nutritional quality indexes. Low values of AI and TI are associated with a low risk of developing thromboembolic events, atherosclerotic processes that lead to cardio‐ and cerebrovascular diseases. Values of AI and TI lower than one are considered low and are associated with health benefits (Chen and Liu 2020). In the samples analyzed, whose AI and TI ranged respectively from 0.53 to 1.03 and from 0.33 to 0.59, only the sample of *R. decussatus* from Capo Peloro lagoon collected in 2024 showed AI greater than 1. The higher saturated fatty acid content found in samples from Capo Peloro lagoon results in higher atherogenicity and thrombogenicity indexes and lower h/H ratio than samples from other lagoons. On the contrary, the lowest levels of atherogenicity and thrombogenicity indices and the highest h/H ratio characterized clams from Sardinia collected in 2024, while in 2023 clams from Oliveri‐Tindari lagoon had the best nutritional indices.

In contrast to AI and TI, higher values of h/H ratio are preferable. This ratio reflects the effect of the FA composition on blood cholesterol levels and, consequently, on cardiovascular health (Bazarsadueva et al. 2021). Its value ranged from 0.85–1.36 in all clam species analyzed. Regarding PUFAs, the n3 series (16.82%–30.67%) predominated over the n6 series (4.43%–9.34%), which determines a n6:n3 ratio less than one, with values ranging from 0.16–0.56. The n6 and n3 fatty acids have enzymatic processes in common and share the same enzymes; however, anti‐inflammatory molecules are synthesized from the n3 series fatty acids and pro‐inflammatory molecules from the n6 series (Mariamenatu and Abdu 2021). The consumption of clams rich in n3 fatty acids promotes the maintenance of a low n6:n3 ratio diet with anti‐inflammatory effects, reducing the risk of many chronic diseases such as cardiovascular disease, inflammation, obesity, diabetes, cancer, and as well as improving mental health (Liput et al. 2021). Bivalves cannot produce n3 and n6 PUFAs on their own to meet their physiological needs, so they obtain these essential fatty acids entirely from their diet. As a consequence, they are good sources of essential fatty acids that are not synthesized in the human body (Romlee et al. 2011). The profitable concentration of EPA and DHA fatty acids typical of seafood is linked to the phytoplankton and algae that are part of their food chain (Gladyshev et al. 2013). Considering the content of fat in the samples analyzed, the content of EPA + DHA ranged from 135.74–298.8 mg/100 g of clams in *R. decussatus* collected in 2023 in Capo Peloro and Santa Gilla lagoons, respectively. This was a very promising result, considering that the reference portion of clams is 150 g and that guidelines recommend an intake of EPA + DHA of 250–500 mg/day (EFSA Panel on Dietetic Products, Nutrition and Allergies (NDA) 2012; Italian Society of Human Nutrition, SINU 2014).

### 3.2 PCA Analysis

**FIGURE 1:**
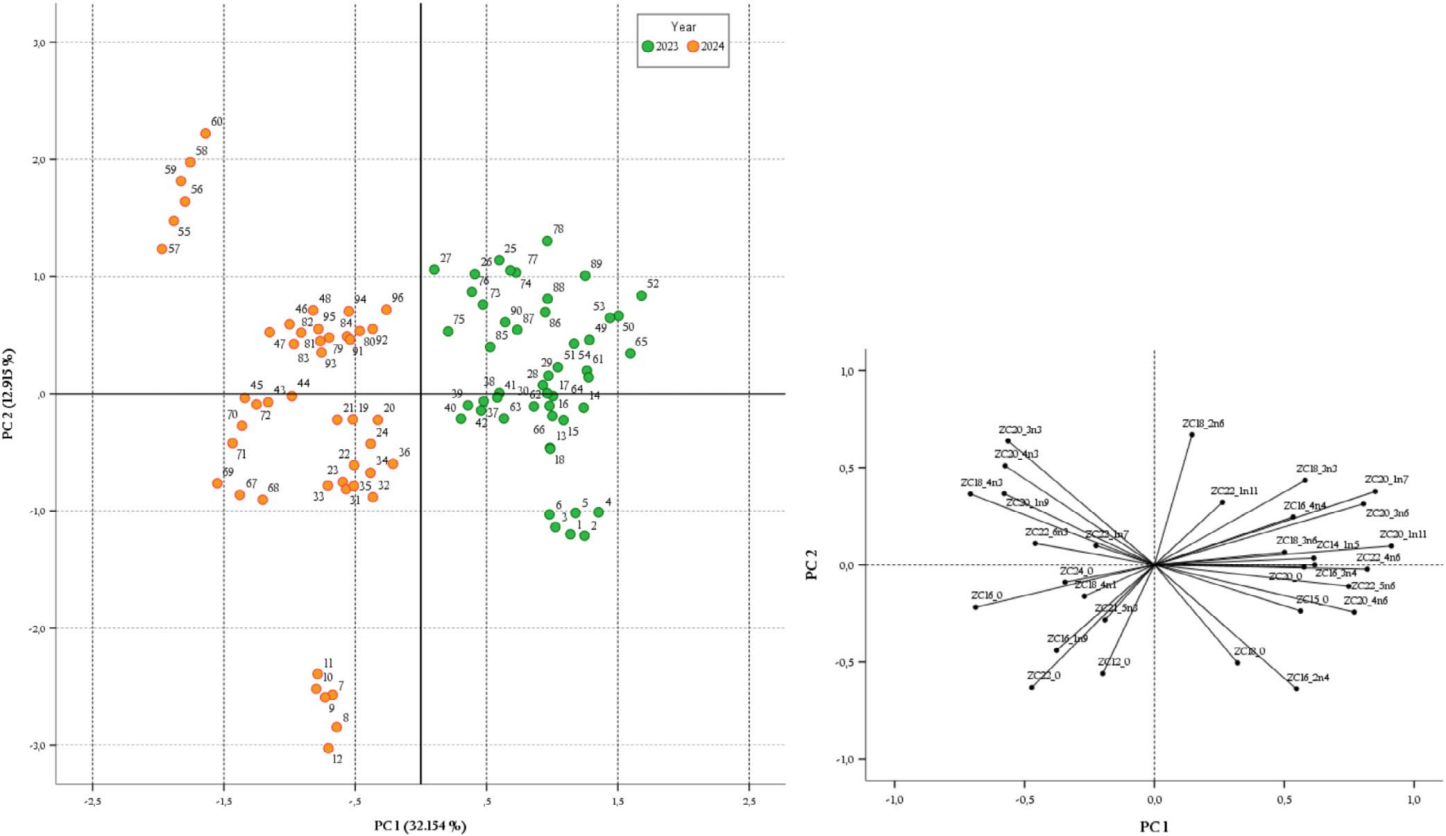
Score plot and loading plot of PC1 and PC2 of experimental data that showed all clam samples differentiated according to the sampling year.

The PCA confirmed the difference between samples collected in 2023 and 2024 and among samples from different TWZs. In this regard, Figure 1 displays the score and the loading plots of the PCA performed to assess the correlation between the samples and the year of sampling. Excluding a few variables that are not statistically different according to the Mann–Whitney test, the new standardized correlation matrix includes 16 cases and 30 variables and was suitable for PCA (KMO value was 0.632). Based on the Kaiser Criterion, 8 principal components were extracted with eigenvalues more than 1.0 (9.646, 3.875, 3.289, 2.557, 2.072, 1.694, 1.270, 1.039) and showed a total variance of 84.81%. The first two principal components (PC1 and PC2) together explain 40.07% of the variance within the system.

Focusing on the score plot, a clear separation between the samples collected in 2023 and 2024 is evident along PC1, indicating distinct differences between the 2 years. By overlaying the score plot and the loading plots, the variables with positive loading on PC1, such as C20:1n11 (0.912), C20:1n7 (0.850) and C22:4n6 (0.819), are associated with the 2023 samples, while those with negative loadings on PC1, such as C18:4n3 (−0.708) and C16:0 (−0.689), are correlated with the 2024 samples.

**FIGURE 2:**
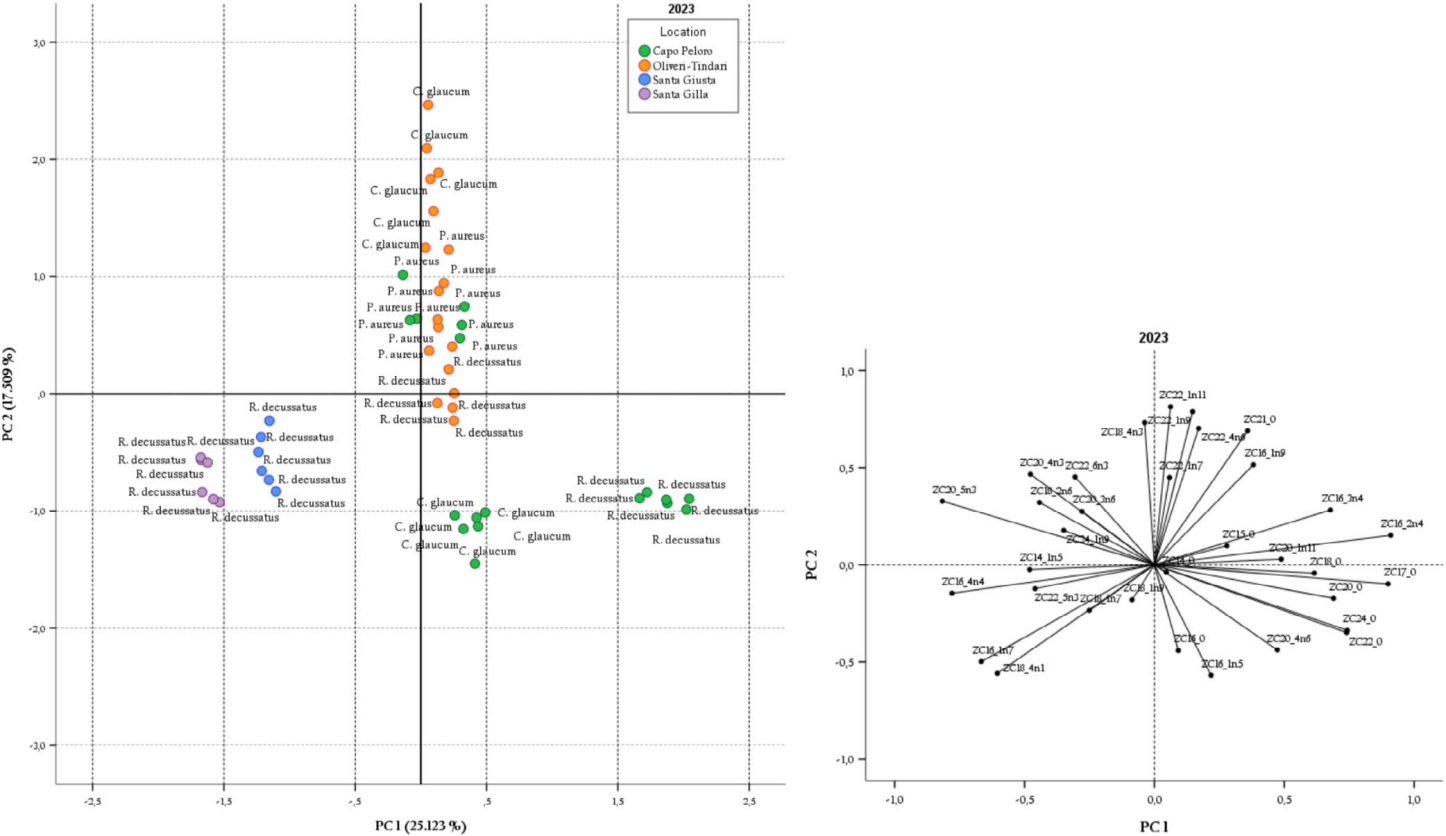
Score plot and loading plot of PC1 and PC2 of experimental data that showed clam samples collected in 2023 differentiated according to the sampling location.

Two further PCAs were performed separately on the samples collected in 2023 and 2024 to analyze their correlation with sampling location. Again, variables not showing statistical differences according to Kruskal‐Wallis one‐way ANOVA were eliminated. The standardized correlation matrices include 48 cases and 33 variables for 2023, and 48 cases and 36 variables for 2024. Figure 2 shows the score and the loading plots of the PCA performed on the 2023 data. Eight principal components with eigen values < 1.0 (8.29, 5.778, 4.652, 4.055, 2.981, 2.186, 1.901, 1.150) are extracted and explain a total variance of 93.92%. PC1 and PC2 explain 42.63% of the variance. Samples collected in 2023 tend to cluster based on the sampling location, except for *P. aureus* from Capo Peloro which overlaps with Oliveri‐Tindari samples, because many of the fatty acids of this species do not show significant differences between the two lagoons. PC1 separates Sardinian from Capo Peloro samples. The latter occupies the IV quadrant of the score plot with strong positive correlations with C16:2 n4 (0.909) and many SFAs such as C17:0 (0.899), C24:0 (0.743) and C22:0 (0.743) in PC1. Comparing the score and loading plots, among Capo Peloro samples, *R. decussatus* separates from *C. glaucum* due to a higher concentration of the above‐mentioned saturates in the first.

Samples from Sardinia are positioned separately in the III quadrant due to the strong influence of C 22:5n3, C16:4n4, and C16:1 n7. This is demonstrated by the overlap of the loading vector and confirmed by their highest concentration.

Oliveri‐Tindari samples cluster distinctly along the PC2, positioning on the positive side of this axis. The variables positively associated with PC2 are C22:1n11 (8.14) and C22:1 n9 (7.88).

**FIGURE 3:**
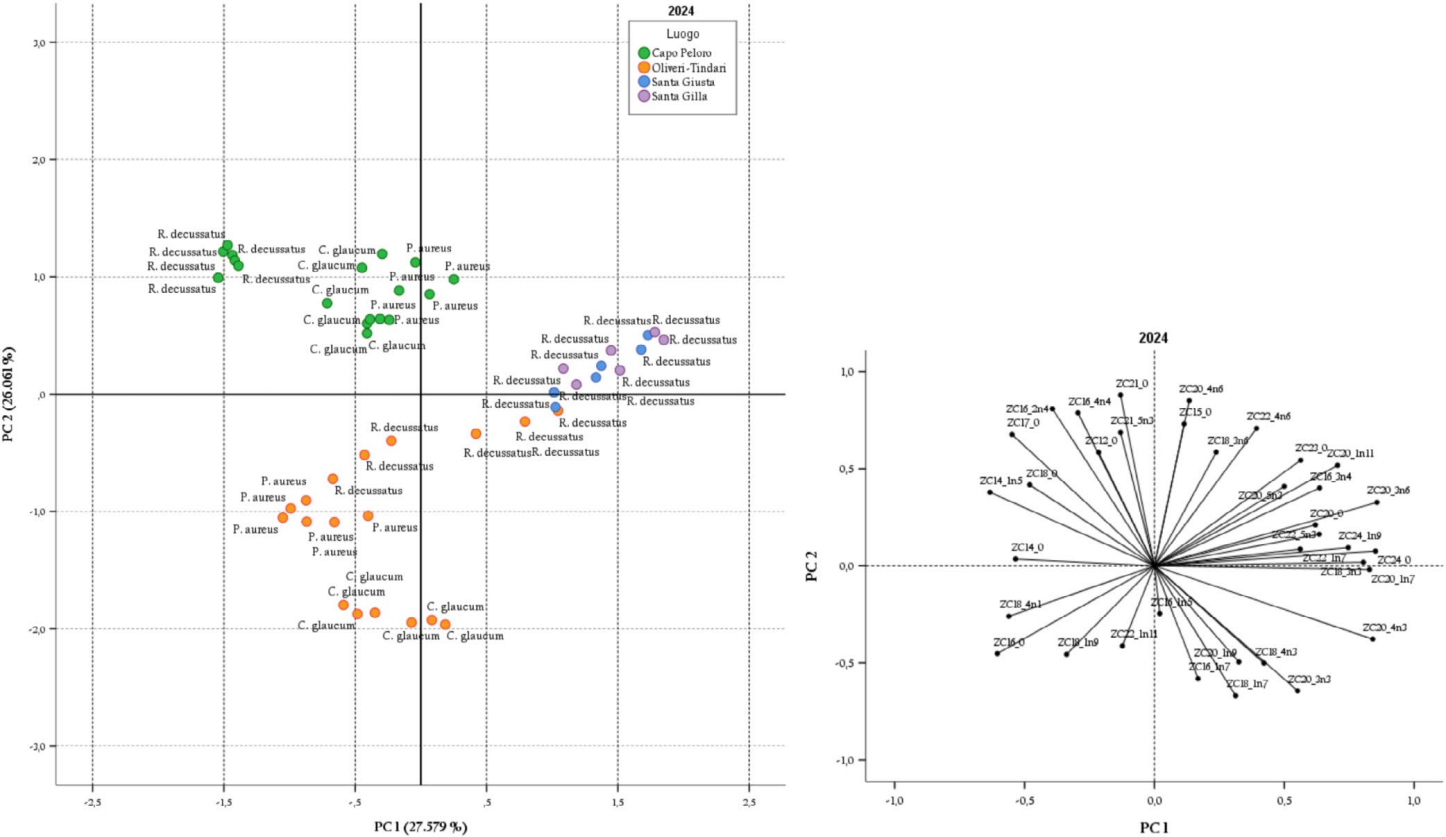
Score plot and loading plot of PC1 and PC2 of experimental data that showed clam samples collected in 2024 differentiated according to the sampling location.

Figure 3 displays the score and the loading plots of the PCA performed on the 2024 data. A total of seven principal components with eigen values < 1.0 (9.92, 9.38, 4.17, 3.53, 2.26, 1.95 and 1.45) are extracted and explain a total variance of 90.75%. PC1 and PC2 explain 53.54% of the variance. PC1 shows a strong positive correlation with C20:3n6 (0.857), C24:0 (8.51), C20:4 n3 (8.40), C20:1n7 (8.28) and C18:3 n3 (0.804). This strong positive correlation results in a separation of the Sardinian and Sicilian samples along PC1, except for a few *R. decussatus* samples from Oliveri‐Tindari. This is confirmed by the fact that the Oliveri‐Tindari samples show fatty acid values that are intermediate between the Capo Peloro and Sardinian samples. PC2 instead separates Capo Peloro from Oliveri‐Tindari samples. By overlaying the loading and score plots, C12:0, C16:2n4, C16:4n4, C17:0, and C18:0 have a great influence on samples from Capo Peloro, while C16:0 and C18:1n9 have an influence on samples from Oliveri‐Tindari.

## 4 Conclusion

The fatty acid profile in all samples followed this trend: SFAs > PUFAs > MUFAs. C16:0 and DHA were the principal fatty acids. In particular, *R. decussatus* and *C. glaucum* from the Capo Peloro lagoon, in both years, stood out for having the highest SFAs content. Regarding PUFAs, the n3 series predominated over the n6 series. Fat showed a high nutritional quality in all samples, as demonstrated by the excellent levels of AI, TI, and h/H.

All clams showed a good content of EPA and DHA as the consumption of a portion of clams (150 g) covers at least 80% of the declared recommended intake, confirming that these foods can be part of a healthy cardioprotective diet.

The PCA showed a clear differentiation between the samples collected in the 2 years, while the differentiation related to sampling location was weaker in both years. Such temporal and spatial differentiation might be linked to both interannual and geographic climatic differences.

It is worth remembering that the presence of inorganic contaminants in the environment significantly influences the fatty acid profile of all clam species.

In this study, the high nutritional quality of the native *R. decussatus*, *C. glaucum* , and *P. aureus* from the Capo Peloro lagoon has been proved, according to the statistical comparison with commercial and non‐commercial samples specifically collected in other differently featured lagoons.

In conclusion, these findings support the claims of Ganzirri as a food product of great historical, cultural, and gastronomic value, while also highlighting their role as a functional, cardioprotective seafood with strong potential for commercial promotion.

## Author Contributions

**Salvatore Giacobbe:** funding acquisition (equal), project administration (equal), visualization (equal). **Giuseppa Di Bella:** conceptualization (equal), supervision (equal), visualization (equal). **Benedetta Sgrò:** data curation (equal), formal analysis (equal), investigation (equal), visualization (equal), writing – original draft (equal), writing – review and editing (equal). **Fabio Scarpa:** data curation (equal), visualization (equal). **Daria Sanna:** data curation (equal), visualization (equal). **Ilenia Azzena:** data curation (equal), visualization (equal). **Marco Casu:** data curation (equal), visualization (equal). **Federica Litrenta:** data curation (equal), software (equal), visualization (equal). **Vincenzo Lo Turco:** conceptualization (equal), resources (equal), visualization (equal).

## Conflicts of Interest

The authors declare no conflicts of interest.

## Supporting information


**Data S1:** Supporting Information.

## Data Availability

The data that support the findings of this study are available from the corresponding author upon reasonable request.
