# Supplementary material for: Comparative Assessment of Fatty Acids in Clams From Capo Peloro Lagoon (Sicily)
Source: Food Sci Nutr. 2025 Sep 12;13(9):e70933. doi: 10.1002/fsn3.70933 (PMC12426908; doi:10.1002/fsn3.70933)
Supplement: Supplementary file 1 — Data S1: Supporting Information. [file FSN3-13-e70933-s001.docx]

**Supplementary Table S1.** Sample collection of clams from Sicilian and Sardinian lagoons.

|  | ***SICILY*** | | | | ***SARDINIA*** | | | |
| --- | --- | --- | --- | --- | --- | --- | --- | --- |
|  | ***Capo Peloro***  ***(Lake Ganzirri )*** | | ***Oliveri-Tindari*** | | ***Santa Gilla*** | | ***Santa Giusta*** | |
| *Winter 2023* |  |  |  |  |  |  |  |  |
|  | 6x500g | *R. decussatus* | 6x500g | *R. decussatus* | 6x500g | *R. decussatus* | 6x500g | *R. decussatus* |
|  | 6x500g | *C. glaucum* | 6x500g | *C. glaucum* |  |  |  |  |
|  | 6x500g | *P. aureus* | 6x500g | *P. aureus* |  |  |  |  |
| *Winter 2024* |  |  |  |  |  |  |  |  |
|  | 6x500g | *R. decussatus* | 6x500g | *R. decussatus* | 6x500g | *R. decussatus* | 6x500g | *R. decussatus* |
|  | 6x500g | *C. glaucum* | 6x500g | *C. glaucum* |  |  |  |  |
|  | 6x500g | *P. aureus* | 6x500g | *P. aureus* |  |  |  |  |
| **TOTAL** | **36x500g** |  | **36x500g** |  | **12x500g** |  | **12x500g** |  |

**Supplementary Table S2.** GC-FID instrument operating conditions.

| **GC-FID** | |
| --- | --- |
| Column | ZB-Wax column (30 m x 0.25 mm, 0.25 μm, Phenomenex, Torrance, CA, USA) |
| Carrier gas flow rate (He) | 30 cm/s |
| Program temperature | 50 °C for 2 min, 3 °C/min until 240 °C, 240°C for 15 min |
| Injector temperature | 240°C |
| Injection volume | 1 µL |
| Injection mode | Splitless with 1:10 split ratio |
| Detector temperatures | 240°C |

**
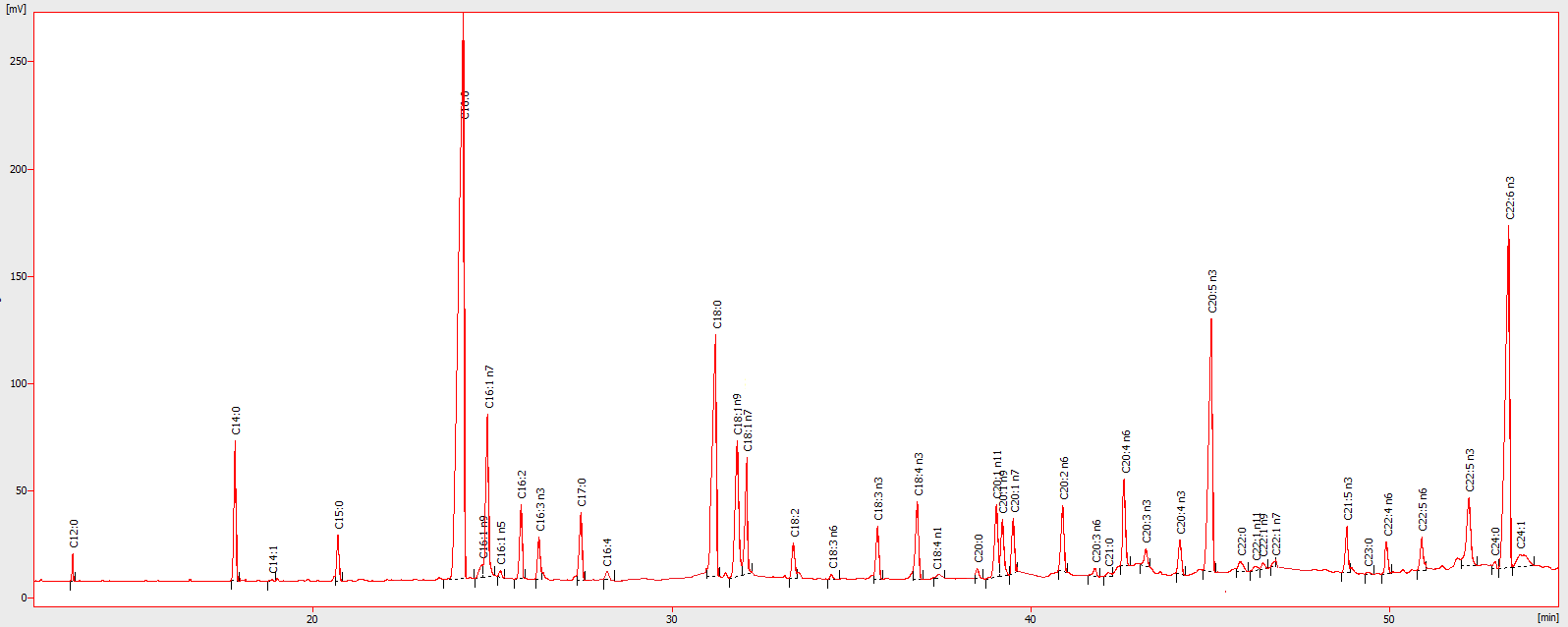
**

**Supplementary Figure S1.** Representative GC-FID chromatogram of fatty acids identified in a *C. glaucum* sample from Ganzirri Lake.

**Supplementary Table S3.** Statistical comparison of total fat and fatty acid profile of clams collected in 2023 and 2024 using Mann-Whitney test.

|  | **Capo Peloro Lagoon (Lake Ganzirri)** | | | **Oliveri-Tindari Lagoon** | | | **Santa Giusta Lagoon** | **Santa Gilla Lagoon** |
| --- | --- | --- | --- | --- | --- | --- | --- | --- |
|  | ***R. decussatus*** | ***C. glaucum*** | ***P. aureus*** | ***R. decussatus*** | ***C. glaucum*** | ***P. aureus*** | ***R. decussatus*** | ***R. decussatus*** |
| **Total fat** | 0.132 | 0.180 | 0.093 | 0.065 | 0.093 | 0.132 | 0.240 | **< 0.05** |
| **C12:0** | **< 0.05** | **< 0.05** | **< 0.05** | **< 0.05** | **< 0.05** | 0.093 | **< 0.05** | 0.589 |
| **C14:0** | **< 0.05** | **< 0.05** | **< 0.05** | **< 0.05** | **< 0.05** | **< 0.05** | **< 0.05** | **< 0.05** |
| **C14:1 n5** | **< 0.05** | **< 0.05** | **< 0.05** | 0.937 | **< 0.05** | **< 0.05** | **< 0.05** | **< 0.05** |
| **C15:0** | **< 0.05** | **< 0.05** | **< 0.05** | 0.394 | **< 0.05** | **< 0.05** | **< 0.05** | **< 0.05** |
| **C16:0** | **< 0.05** | 0.093 | 1.00 | **< 0.05** | **< 0.05** | **< 0.05** | 0.818 | 0.485 |
| **C16:1 n9** | **< 0.05** | **< 0.05** | **< 0.05** | **< 0.05** | **< 0.05** | **< 0.05** | **< 0.05** | **< 0.05** |
| **C16:1 n7** | 0.818 | **< 0.05** | **< 0.05** | **< 0.05** | **< 0.05** | **< 0.05** | **< 0.05** | **< 0.05** |
| **C16:1 n5** | 0.065 | **< 0.05** | **< 0.05** | **< 0.05** | 0.132 | **< 0.05** | 0.132 | **< 0.05** |
| **C16:2 n4** | 0.132 | 1.000 | 0.310 | **< 0.05** | **< 0.05** | **< 0.05** | **< 0.05** | 0.485 |
| **C16:3 n4** | **< 0.05** | **< 0.05** | **< 0.05** | **< 0.05** | **< 0.05** | **< 0.05** | **< 0.05** | **< 0.05** |
| **C17:0** | 0.132 | 0.240 | 0.065 | 0.065 | 0.065 | **< 0.05** | 0.485 | **< 0.05** |
| **C16:4 n4** | **< 0.05** | 0.065 | **< 0.05** | **< 0.05** | **< 0.05** | **< 0.05** | **< 0.05** | **< 0.05** |
| **C18:0** | 0.132 | 1.000 | **< 0.05** | **< 0.05** | **< 0.05** | 0.485 | 0.180 | 0.818 |
| **C18:1 n9** | **< 0.05** | **< 0.05** | 0.093 | 0.180 | **< 0.05** | 0.093 | **< 0.05** | **< 0.05** |
| **C18:1 n7** | **< 0.05** | 0.394 | **< 0.05** | 0.937 | **< 0.05** | 0.093 | 0.093 | 0.240 |
| **C18:2 n6** | 0.240 | 0.485 | **< 0.05** | 0.093 | 0.093 | **< 0.05** | **< 0.05** | 0.065 |
| **C18:3 n6** | **< 0.05** | 0.699 | 0.485 | **< 0.05** | **< 0.05** | **< 0.05** | 0.180 | 0.093 |
| **C18:3 n3** | **< 0.05** | **< 0.05** | **< 0.05** | **< 0.05** | 1.000 | **< 0.05** | 0.485 | 0.180 |
| **C18:4 n3** | **< 0.05** | **< 0.05** | 0.065 | **< 0.05** | **< 0.05** | **< 0.05** | **< 0.05** | **< 0.05** |
| **C18:4 n1** | **< 0.05** | **< 0.05** | **< 0.05** | **< 0.05** | **< 0.05** | **< 0.05** | **< 0.05** | **< 0.05** |
| **C20:0** | **< 0.05** | 1.000 | **< 0.05** | 1.000 | **< 0.05** | **< 0.05** | 0.394 | 0.180 |
| **C20:1 n11** | **< 0.05** | **< 0.05** | **< 0.05** | **< 0.05** | **< 0.05** | **< 0.05** | **< 0.05** | **< 0.05** |
| **C20:1 n9** | **< 0.05** | **< 0.05** | **< 0.05** | **< 0.05** | **< 0.05** | **< 0.05** | **< 0.05** | **< 0.05** |
| **C20:1 n7** | **< 0.05** | **< 0.05** | **< 0.05** | **< 0.05** | **< 0.05** | **< 0.05** | **< 0.05** | **< 0.05** |
| **C20:2 n6** | **< 0.05** | 0.589 | 0.180 | **< 0.05** | 0.065 | **< 0.05** | **< 0.05** | **< 0.05** |
| **C20:3 n6** | **< 0.05** | **< 0.05** | **< 0.05** | 0.065 | **< 0.05** | **< 0.05** | **< 0.05** | **< 0.05** |
| **C21:0** | **< 0.05** | **< 0.05** | 0.093 | 0.065 | **< 0.05** | **< 0.05** | **< 0.05** | **< 0.05** |
| **C20:4 n6** | **< 0.05** | **< 0.05** | 0.394 | **< 0.05** | **< 0.05** | **< 0.05** | **< 0.05** | **< 0.05** |
| **C20:3 n3** | **< 0.05** | 0.589 | 1.000 | 0.485 | **< 0.05** | **< 0.05** | **< 0.05** | **< 0.05** |
| **C20:4 n3** | 0.699 | **< 0.05** | 0.310 | **< 0.05** | **< 0.05** | **< 0.05** | **< 0.05** | **< 0.05** |
| **C20:5 n3** | **< 0.05** | **< 0.05** | **< 0.05** | 0.818 | **< 0.05** | **< 0.05** | **< 0.05** | 0.240 |
| **C22:0** | 0.485 | **< 0.05** | **< 0.05** | **< 0.05** | **< 0.05** | **< 0.05** | **< 0.05** | **< 0.05** |
| **C22:1 n11** | **< 0.05** | **< 0.05** | **< 0.05** | **< 0.05** | **< 0.05** | **< 0.05** | **< 0.05** | 0.093 |
| **C22:1 n9** | **< 0.05** | **< 0.05** | 0.699 | 0.699 | **< 0.05** | 0.818 | **< 0.05** | 0.818 |
| **C22:1 n7** | **< 0.05** | 1.000 | **< 0.05** | **< 0.05** | 0.818 | **< 0.05** | **< 0.05** | **< 0.05** |
| **C21:5 n3** | **< 0.05** | **< 0.05** | **< 0.05** | **< 0.05** | 0.065 | **< 0.05** | **< 0.05** | 0.310 |
| **C23:0** | **< 0.05** | **< 0.05** | 0.240 | **< 0.05** | **< 0.05** | **< 0.05** | **< 0.05** | **< 0.05** |
| **C22:4 n6** | **< 0.05** | **< 0.05** | 1.000 | **< 0.05** | **< 0.05** | **< 0.05** | **< 0.05** | **< 0.05** |
| **C22:5 n6** | **< 0.05** | **< 0.05** | 0.699 | 0.589 | **< 0.05** | **< 0.05** | **< 0.05** | 0.180 |
| **C22:5 n3** | **< 0.05** | 0.310 | **< 0.05** | 0.394 | 0.394 | 0.132 | **< 0.05** | **< 0.05** |
| **C24:0** | **< 0.05** | **< 0.05** | **< 0.05** | **< 0.05** | **< 0.05** | **< 0.05** | **< 0.05** | **< 0.05** |
| **C22:6 n3** | 0.394 | **< 0.05** | **< 0.05** | 0.589 | 0.093 | **< 0.05** | 0.180 | 0.180 |
| **C24:1 n9** | **< 0.05** | 1.000 | **< 0.05** | **< 0.05** | **< 0.05** | **< 0.05** | **< 0.05** | **< 0.05** |
| **SFA** | **< 0.05** | 0.180 | 0.485 | **< 0.05** | **< 0.05** | **< 0.05** | 0.818 | 0.093 |
| **MUFA** | **< 0.05** | **< 0.05** | **< 0.05** | **< 0.05** | 0.065 | 0.485 | **< 0.05** | 0.065 |
| **PUFA** | **< 0.05** | **< 0.05** | **< 0.05** | **< 0.05** | **< 0.05** | **< 0.05** | **< 0.05** | 0.589 |
| **∑ n6** | **< 0.05** | **< 0.05** | 0.065 | **< 0.05** | **< 0.05** | **< 0.05** | **< 0.05** | 0.240 |
| **∑ n3** | **< 0.05** | **< 0.05** | **< 0.05** | 0.240 | **< 0.05** | 0.310 | **< 0.05** | **< 0.05** |
| **n6:n3 ratio** | **< 0.05** | **< 0.05** | **< 0.05** | **< 0.05** | **< 0.05** | **< 0.05** | **< 0.05** | **< 0.05** |
| **AI** | **< 0.05** | 0.699 | **< 0.05** | **< 0.05** | **< 0.05** | **< 0.05** | 0.394 | 0.180 |
| **TI** | **< 0.05** | **< 0.05** | **< 0.05** | **< 0.05** | 0.310 | **< 0.05** | **< 0.05** | 0.310 |
| **h/H** | **< 0.05** | 0.394 | **< 0.05** | **< 0.05** | 0.004 | **< 0.05** | 0.818 | 0.937 |
| **EPA+DHA** | **< 0.05** | **< 0.05** | **< 0.05** | 0.240 | 0.132 | 0.093 | **< 0.05** | 0.180 |


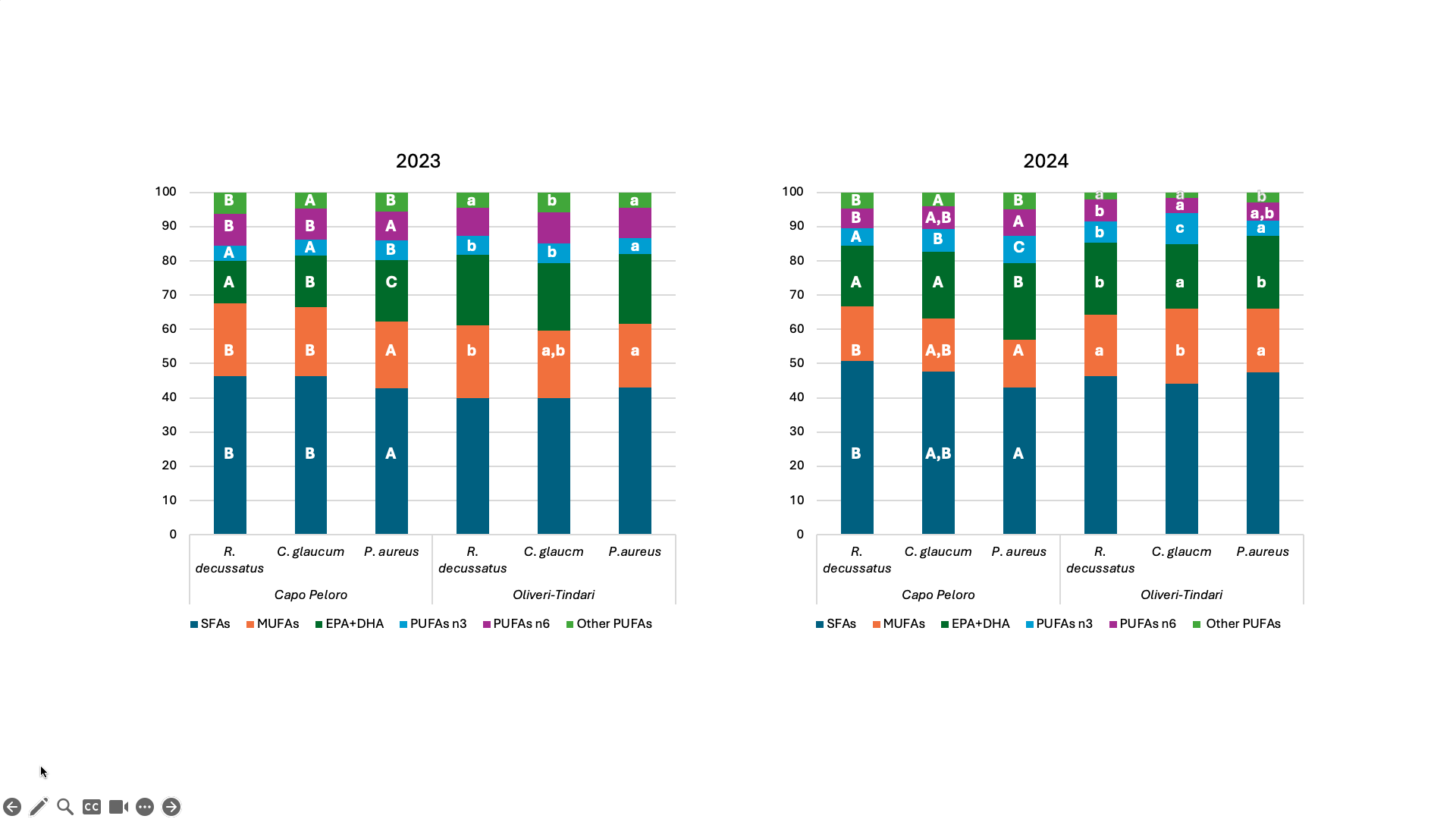


**Supplementary Figure S2.** Graphical and statistical comparison of fatty acid profile. Different letters in the column of the same colour indicate statistically different results (p < 0.05) obtained by Kruskal–Wallis test for different species collected in the same lagoon in 2023 (left) and 2024 (right).
